# Association of serum selenium levels with diabetic retinopathy: NHANES 2011–2016

**DOI:** 10.3389/fmed.2025.1546214

**Published:** 2025-04-04

**Authors:** Xi Chen, Mei Sun, Zhenzhen Gu, Xiaofeng Hao, Like Xie

**Affiliations:** Eye Hospital, China Academy of Chinese Medical Sciences, Beijing, China

**Keywords:** selenium, diabetic retinopathy, NHANES, epidemiology, cross-sectional study

## Abstract

**Background:**

Several studies have established a clear link between serum selenium levels and various health outcomes. However, to date, only a few studies have found an association between serum selenium levels and diabetic retinopathy (DR). The exact link between them is unclear. We collected data from different patient populations.

**Methods:**

Data from 645 adults, collected through the National Health and Nutrition Examination Survey (NHANES) between 2011 and 2016, were analyzed. The association between serum selenium levels and the incidence of DR was assessed using binary logistic regression. Subgroup analysis, smoothed curve-fitting analysis, and propensity score weighting were used to investigate the association further.

**Results:**

According to the multivariate analysis, there was no statistically significant linear association between serum selenium levels and the probability of developing DR (*p* > 0.05). Segmented regression analysis, however, showed that the chance of developing DR was considerably lower when selenium levels reached the threshold of 106.8 μg/L (OR = 0.88, *p* = 0.0107).

**Conclusion:**

A U-shaped curve represents the link between serum selenium levels and DR. The incidence of DR is elevated in individuals with serum selenium levels that are either higher or lower than the optimal range.

## 1 Introduction

One of the most common microvascular complications that affect people with diabetes is diabetic retinopathy (DR) (1). Characterized mainly by retinal microangiopathy, diabetic retinopathy is a major cause of adult blindness worldwide (2). DR usually appears in those with poor glycemic control and a long history of diabetes. Statistics show that approximately 34.6% of individuals with diabetes worldwide are affected by DR, and approximately 10% of these patients experience severe forms of the condition, leading to significant vision impairment (3). Diabetes affects an estimated 537 million people worldwide in 2021. Diabetes prevalence continues to climb, with the population expected to reach 784 million by 2045 (4). The global burden of DR has grown significantly, with diabetes-related visual loss estimated to cost $500 million by 2050. It is therefore vital to combat DR. However, it is really tough (5).

Selenium is a key trace element found in many foods that is crucial for human health and has been demonstrated to reduce the incidence of certain cancers (6). After absorption, selenium is incorporated into several selenoproteins, which mediate its biological actions. Some of the biological actions of selenium, including promoting apoptosis, inhibiting vascular endothelial growth factor, neoangiogenesis, and matrix metalloproteinases, can hinder cancer spread by preventing tumor growth and metastasis. While selenium is essential for good health, an excessive intake can be detrimental. These effects can also be toxic to healthy tissues and disrupt normal cellular functions.

Some research indicates that minerals could aid in the prevention of DR (7). The development of DR is heavily influenced by oxidative stress and chronic inflammation caused by hyperglycemia. Selenium, an essential antioxidant trace element, has been shown to mitigate oxidative stress and inflammation by enhancing glutathione peroxidase activity (8). Consequently, both selenium deficiency and excess may exacerbate oxidative stress and inflammation, potentially compromising the health of retinal microvasculature (9). However, the connection between serum selenium levels and the prevalence of DR remains unclear. Some studies suggest that people with diabetes tend to have lower serum selenium levels than healthy individuals (10). In contrast, some studies have suggested that greater serum selenium levels are associated with an increased risk of developing type 2 diabetes (11, 12). This discrepancy could be attributed to the dual role of selenium in both regulating insulin signaling pathways and modulating oxidative stress (13).

In conclusion, whereas heavy metals have been linked to an increased risk of developing DR, the relationship between selenium and DR remains uncertain. The purpose of this study was to investigate the association between serum selenium levels and the prevalence of DR by analyzing data from the 2011–2016 NHANES. The findings may shed light on the potential role of selenium in preventing and managing DR.

## 2 Materials and methods

### 2.1 Study participants

**Figure 1:**
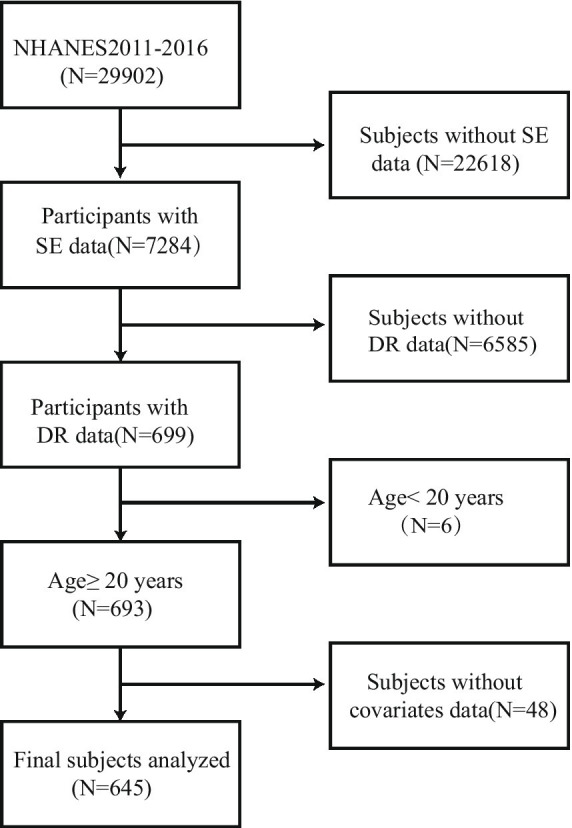
Diagrammatic representation of patient selection. NHANES, National Health and Nutrition Examination Survey. DR, diabetic retinopathy.

The NHANES is a nationally representative transect survey. It can provide objective data about the population’s nutritional and health problems. The NHANES collects data through a multistage random sample. All participants were subjected to comprehensive measurements and completed standardized interview questionnaires. We analyzed NHANES data from 2011 to 2016. Of the 29,902 participants in NHANES 2011–2016, we excluded those with missing serum selenium screening data (*N* = 22,618) and no information on DR (*N* = 6,585). Furthermore, minor participants (*n* = 6) and those lacking complete data on other covariates (*n* = 48) were excluded. Finally, the final number of subjects included in the analysis was 645. Figure 1 depicts the participant selection process.

### 2.2 Diabetic retinopathy assessment

First, diabetes screening was performed, and then, screening for DR in the diabetic population was carried out. The diagnosis of diabetes among participants was established based on the fulfillment of any of the following conditions: (1) hemoglobin A1c (HbA1c) levels equal to or exceeding 6.5%; (2) fasting plasma glucose (FPG) concentrations of 7 mmol/L or higher; (3) a 2-h plasma glucose value of 11.1 mmol/L or greater during an oral glucose tolerance test; (4) self-reported information indicating a prior diagnosis of diabetes by a healthcare professional; and (5) ongoing treatment with insulin or other antidiabetic medications. DR was assessed using a two-point self-report question, where participants indicated whether a doctor had informed them that diabetes had been affecting their eyes (DIQ080). A “yes” response resulted in a DR diagnosis.

### 2.3 Determination of serum selenium

A physician performed venipuncture to collect serum samples, which were subsequently processed and analyzed at the Centers for Disease Control and Prevention (CDC) laboratory. The samples were cryopreserved. Following dilution, the blood samples were analyzed using ICP-DRC-MS. Samples must be homogenously mixed to avoid the formation of clots, which could otherwise affect the analytical results. The method is capable of performing highly sensitive multi-element analyses. The method ensures the accurate determination of trace elements, including zinc, copper, and selenium. All results were above the acceptable lower limit of detection.

### 2.4 Covariate assessment

Demographic data, such as sex, age, race, marital status, education level, and economic status, were included. BMI was divided into three distinct groups: normal or underweight (less than 25.0 kg/m^2^), overweight (between 25.0 and 29.9 kg/m^2^), and obese (30.0 kg/m^2^ or higher). Lifestyle factors were assessed using a self-report questionnaire. Participants were classed as either non-drinkers or drinkers based on their self-reported consumption of more than 12 alcoholic beverages over the course of their lifetime. Smokers were divided into two groups: never smokers and smokers, based on whether they had smoked fewer than 100 cigarettes in their lifetime. Participants were considered to have high blood pressure if a doctor had informed them of the condition, if they were taking blood pressure medication, or if their blood pressure was higher than normal.

### 2.5 Statistical analysis

To achieve the desired results, we employed both weighted and variance estimation methods. Weighted multivariate logistic regression models were employed. Continuous variables were assessed using independent *t*-tests, and categorical variables were evaluated with the chi-square tests. Logistic regression models were employed to examine the relationship between serum selenium levels and DR. Subgroup analyses were conducted using covariates, and further subgroup analyses were performed using stratified multiple regression analysis. Differences across subgroups were investigated, as well as potential interaction effects. All analyses were conducted using R (version 4.1.1) and EmpowerStats (version 4.2). All analyses were performed using regression models, with a significance threshold set at a *p*-value of 0.05, where *p*-values of 0.05 or lower were considered statistically significant.

## 3 Results

### 3.1 Study population characteristics

As shown in Table 1, the study divided participants into two groups according to whether they had DR or not. In the analysis of factors associated with DR, the study found that all variables did not show statistically significant differences.

**Table 1:** Baseline characteristics of participants.

Characteristic	No DR	DR	*P*-value
N	502	143	
Age (years)	61.2 ± 12.8	61.8 ± 11.2	0.614
Sex (*N*, %)			0.066
Male	261 (50.2)	84 (62.9)	
Female	241 (49.8)	59 (37.1)	
Race (*N*, %)			0.179
Mexican American	85 (8.8)	24 (9.5)	
Non-Hispanic White	173 (66.6)	46 (58.6)	
Non-Hispanic Black	132 (13.0)	34 (13.5)	
Other Race	112 (11.6)	39 (18.3)	
Marital status (*N*, %)			0.938
Unmarried or other	299 (61.9)	85 (61.4)	
Married or living with a partner	203 (38.1)	58 (38.6)	
Education (*N*, %)			0.115
Less than high school	172 (22.6)	52 (23.0)	
High school	113 (24.8)	42 (36.4)	
More than high school	217 (52.6)	49 (40.6)	
Smoked at least 100 cigarettes in life (*N*, %)			0.754
Yes	257 (53.8)	71 (56.0)	
No	245 (46.2)	72 (44.0)	
Alcohol usage (*N*, %)			0.689
Yes	336 (72.3)	91 (70.0)	
No	166 (27.7)	52 (30.0)	
BMI (*N*, %)			0.584
Normal or low-weight	65 (10.0)	23 (13.4)	
Overweight	136 (26.8)	43 (29.6)	
Obese	301 (63.3)	77 (56.9)	
Economic situation (*N*, %)			0.467
Below poverty	119 (15.8)	39 (21.4)	
Poverty or above	334 (76.8)	93 (72.3)	
Unclear	49 (7.4)	11 (6.3)	
Hypertension (*N*, %)			0.430
Yes	374 (74.1)	106 (70.2)	
No	128 (25.9)	37 (29.8)	
Cu (ug/dL)	122.6 ± 29.6	121.1 ± 28.3	0.401
Zn (ug/dL)	83.2 ± 15.5	78.9 ± 13.1	0.131

### 3.2 Association between serum selenium and DR

The regression analysis provided in Table 2 revealed that there was no statistically significant overall association between serum selenium levels and DR (*p* > 0.05). However, patients with selenium levels in the third quartile (Q3) exhibited a significantly reduced risk of DR (*p* < 0.01). The effect of extremely low (Q1, Q2) or high (Q4) selenium levels was not statistically significant. The trend tests indicated that there was no statistically significant linear relationship between selenium levels and the risk of developing DR. This indicates that selenium may exert a protective effect at moderate levels, potentially exhibiting a ‘U-shaped’ relationship.

**Table 2:** Relationship between serum selenium and DR.

Variables	Model 1^a^ β (95% CI)	Model 2^b^ β (95% CI)	Model 3^c^ β (95% CI)
	*P*-value	*P*-value	*P*-value
Selenium	0.99 (0.98, 1.01) 0.2803	0.99 (0.98, 1.00) 0.1765	0.99 (0.98, 1.00) 0.2076
Selenium quartile			
Q1	Ref.	Ref.	Ref.
Q2	0.68 (0.34, 1.35) 0.2791	0.61 (0.29, 1.26) 0.1862	0.59 (0.26, 1.36) 0.2267
Q3	0.36 (0.19, 0.69) 0.0036	0.32 (0.16, 0.63) 0.0022	0.35 (0.17, 0.73) 0.0097
Q4	0.87 (0.46, 1.66) 0.6859	0.70 (0.37, 1.33) 0.2841	0.81 (0.42, 1.56) 0.5286
P for trend	0.4297	0.1882	0.4011

a Non-adjusted model: no adjustment for covariates.

b Adjusted model: adjusted for sex, age, and race.

c Adjusted model: adjusted for all covariates.

### 3.3 Identification of non-linear relationship

**Figure 2:**
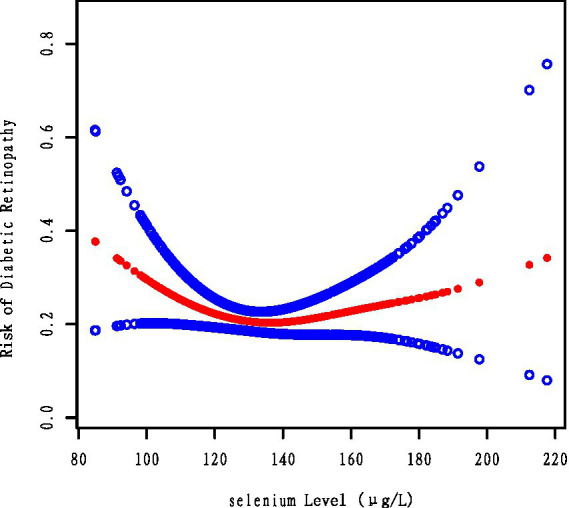
Smooth curve fitting analysis demonstrates the association between SE and the prevalence of DR.

As illustrated by the smoothed curve fit in Figure 2 and the segmented regression analysis in Table 3, serum selenium levels exhibited a U-shaped relationship with the risk of DR. A one-unit increase in selenium levels significantly reduced the risk of DR when selenium levels were below the threshold of 106.8 μg/L (OR = 0.88, *p* = 0.0107). Conversely, changes in selenium level above the turning point were observed to have no significant effect on the risk of DR (OR = 1.00, *p* = 0.5630). A linear log-likelihood ratio test (LRT, *p* = 0.009) provided further support for the superiority of the segmented model over the unilinear model, indicating that selenium may play a more significant protective role at lower levels.

**Table 3:** Analysis of threshold effects of serum selenium and DR.

Models	Per-unit increase		Per-SD increase	
	OR (95%CI)	P-value	OR (95%CI)	P-value
Model 1				
One line effect	1.00 (0.99, 1.01)	0.7019	0.96 (0.78, 1.19)	0.7019
Model 2				
Turning point (K)	106.8		−1.33	
< K	0.88 (0.80, 0.97)	0.0107	0.07 (0.01, 0.53)	0.0108
> K	1.00 (0.99, 1.01)	0.5630	1.07 (0.86, 1.32)	0.5658
95% CI for turning point	−1.69, −0.99		−1.69, −0.99	
*P*-value for LRT test		0.009		0.009

### 3.4 Subgroup analyses

As demonstrated in the stratified analysis presented in Table 4, the risk of DR was associated with age, gender, education level, and hypertension. The risk of DR was observed to be slightly lower in the older age group of 67–80 years (*p* = 0.0268), men (*p* = 0.0244), those with a high school education (*p* = 0.0424), and hypertension (*p* = 0.0187). No significant associations were found between race and marital status, and none of the interaction effects between these variables were statistically significant. This suggests that the independent effects of these factors on DR risk are limited.

**Table 4:** Subgroup analysis of serum selenium and DR.

Variables	OR (95% CI)	P-value	P-interaction
Age			0.2257
21–56	1.00 (0.99, 1.02)	0.6094	
57–66	1.00 (0.98, 1.02)	0.8911	
67–80	0.98 (0.96, 1.00)	0.0268	
Sex			0.9992
Male	0.98 (0.97, 1.00)	0.0244	
Female	1.00 (0.99, 1.02)	0.6276	
Race			0.4715
Mexican American	0.99 (0.96, 1.02)	0.5075	
Non-Hispanic White	0.99 (0.98, 1.01)	0.2928	
Non-Hispanic Black	0.99 (0.96, 1.01)	0.303	
Other race	1.00 (0.98, 1.02)	0.8912	
Education			0.2851
Less than high school	1.00 (0.99, 1.02)	0.5197	
High school	0.98 (0.96, 1.00)	0.0424	
More than high school	1.00 (0.98, 1.01)	0.6745	
Marital status			0.9907
Unmarried or other	1.00 (0.99, 1.01)	0.7782	
Married or living with a partner	0.99 (0.98, 1.01)	0.2027	
Hypertension			0.0944
Yes	0.99 (0.97, 1.00)	0.0187	
No	1.01 (1.00, 1.03)	0.0878	

## 4 Discussion

This study presents the first evidence linking selenium levels to the risk of DR. Moreover, by controlling for confounders did not change the association between selenium levels and DR. Studies have shown a U-shaped correlation between serum selenium levels and overall DR risk. The results of the segmented regression analysis indicated that the risk of DR was significantly reduced when selenium levels were at the tipping point of 106.8 (OR = 0.88, *p* = 0.0107). These findings indicate that maintaining selenium levels approximately 106.8 μg/L may confer a protective effect. The combined effect of selenium was found to exhibit a ‘U-shaped’ characteristic when analyzed using a smoothed curve-fitting technique. Our findings indicated that higher serum selenium levels were associated with an increased risk of DR. Existing studies do find that excessive levels of selenium may increase the risk of DR (14). In addition, zinc levels were significantly lower in the DR group than in the control group (*p* = 0.003). These results further support the hypothesis that micronutrients may influence the development of DR.

Studies have analyzed the association between serum heavy metals (e.g., manganese, lead, and cadmium) and DR (15). Manganese was found to be significantly negatively correlated with DR. Nevertheless, no notable correlation was observed between heavy metals, including selenium, and the occurrence of DR. In contrast, the present study focused on the trace element selenium (14). We found a strong correlation between serum selenium levels and the risk of developing DR. The reason for this discrepancy may be the lack of serum selenium data for 2017–2020 in the NHANES database. Moreover, the scope of their study is 2011–2020. The treatment of missing data in their study may have contributed to the results. We excluded populations with missing serum selenium data. This makes our results relatively more reliable, despite the differences in analytical methodology and research focus between the two studies. However, both studies highlighted the potential involvement of trace elements in the pathogenesis of DR. One study has shown that heavy metals are associated with the risk of DR. However, this study did not include selenium (16). Our study investigated the relationship between serum selenium and DR. Another study showed that selenium can reduce oxidative damage (17). This has implications for the prevention and treatment of DR. Other clinical studies have found that selenium does have an effect on the development of DR (18, 19). However, their study did not look at the effect of specific levels of selenium in combating DR. Our findings indicated that selenium levels below the tipping point were associated with a significantly lower risk of DR. The data revealed a U-shaped association between selenium levels and DR. In addition, a potential risk factor for DR was identified as zinc deficiency. This result is consistent with existing studies (20, 21). Existing studies have found that zinc improves diabetic cataracts by modulating lens protein and polyol pathways (22). However, it is not yet known exactly how to improve DR. It is recommended that future studies should further investigate the specific roles and mechanisms of these elements in the prevention and treatment of DR (23).

Selenium affects DR through several mechanisms. First, selenium is an important component of selenoproteins, such as glutathione peroxidase and catalase. They play a key role in neutralizing reactive oxygen species and reducing oxidative stress (24). Oxidative stress is an important cause of DR pathogenesis. By enhancing the activity of these antioxidant enzymes, selenium helps reduce oxidative stress and attenuates retinal cell damage (25). Second, diabetes causes the body to be in a chronic low-grade inflammatory state for a long time. It increases the incidence of DR. Selenium modulates the inflammatory response by reducing the production of pro-inflammatory cytokines and tumor necrosis factor-*α* (TNF-α) (26). This helps reduce inflammatory damage to retinal microvessels and neural tissue. Third, selenium has been shown to enhance vascular endothelial cell function by reducing endothelial dysfunction (27). This reduces vascular leakage and neovascularization, thereby protecting the retinal vasculature to better protect visual function. Fourth, emerging evidence suggests that selenium may play a role in glucose metabolism and insulin signaling. Hyperglycemia is a major cause of DR. By improving insulin sensitivity and reducing hyperglycemia, selenium may indirectly reduce the risk of DR onset and progression (28, 29). Fifth, retinal fibrosis is a late complication of DR that can lead to vision loss (30). Selenium has been shown to inhibit the activation of transforming growth factor-*β* (TGF-β) and reduce the deposition of extracellular matrix components such as collagen and fibronectin (31). By blocking retinal fibrosis, selenium may be able to reduce fibrosis in the retina and may play a role in slowing the advancement of DR. Finally, recent studies have identified a potential role for selenium in epigenetic modifications, such as DNA methylation and histone acetylation. This may affect the expression of genes involved in oxidative stress, inflammation, and angiogenesis (32). These epigenetic effects may further contribute to the protective effects of selenium against DR. In conclusion, selenium has multifaceted mechanisms for the prevention and treatment of DR. Further studies are needed to fully elucidate these mechanisms in the future and explore the potential for selenium intervention in clinical settings.

However, it is important to acknowledge several limitations in this study. First, the cross-sectional design prevents establishing a causal link between blood selenium levels and the development of DR. Second, the absence of longitudinal data precludes the determination of changes in selenium levels over time and their potential correlation with the progression of DR. It is recommended that future studies should incorporate longitudinal data to further validate the causal relationship between selenium levels and DR. Third, the outcome measures relied on self-reported histories of DR, which may not be entirely reliable. Fourth, the study sample was limited due to the substantial amount of missing data on DR and selenium in the NHANES database. Future studies with larger sample sizes are still needed to validate our findings. Finally, the study took into account as many confounding factors as possible. However, there are still unmeasured variables that may affect the results. We hope that future studies will collect relevant data (e.g., diet and lifestyle) more comprehensively to reduce bias.

## 5 Conclusion

In conclusion, the results of this study reveal a U-shaped association between serum selenium levels and DR, suggesting that selenium supplementation should be balanced to avoid excessive intake. Moderate selenium consumption has shown potential benefits in preventing DR. In addition, the findings highlight the possible role of both serum selenium and zinc in DR prevention. Future longitudinal studies are needed to better understand the underlying mechanisms of the effects of selenium, which could help develop personalized prevention and intervention strategies for DR.

## Data Availability

The raw data supporting the conclusions of this article will be made available by the authors, without undue reservation.
